# Malignant cerebral edema secondary to herpes simplex virus encephalitis requiring decompressive hemicraniectomy

**DOI:** 10.1016/j.radcr.2026.09.041

**Published:** 2026-10-05

**Authors:** Evans Konney, Jonathan R. Forrest, Tapan Thaker, Gnan Thakore

**Affiliations:** a Department of Medicine, San Antonio Military Medical Center, San Antonio, TX, USA; b Department of Radiology, Loyola University Medical Center, Maywood, IL, USA; c Clinical Neuroscience Institute, Wright State University, Dayton, OH, USA

**Keywords:** Herpes simplex encephalitis, Malignant cerebral edema, Craniectomy, Magnetic resonance imaging, Computed tomography, Radiology

## Abstract

Herpes simplex encephalitis (HSE) is a severe viral infection of the central nervous system that most commonly affects the temporal lobes and limbic structures. Although most cases are managed medically with intravenous acyclovir, rare cases can rapidly progress to malignant cerebral edema, mass effect, and neurologic deterioration requiring surgical intervention. We report the case of a 57-year-old woman presenting with altered mental status, headache, vomiting, myalgias, and focal neurologic deficits. Initial computed tomography (CT) and CT angiography of the head and neck were unremarkable, but magnetic resonance imaging (MRI) demonstrated diffusion restriction involving the right mesial temporal lobe and insular cortex. Cerebrospinal fluid testing later confirmed HSE. Despite treatment with intravenous acyclovir, the patient developed worsening right hemispheric edema with progressive midline shift, requiring external ventricular drain placement followed by emergent right decompressive hemicraniectomy. She subsequently improved without persistent focal neurologic deficits and later underwent cranioplasty. Our case highlights the importance of early recognition with neuroimaging, close neurologic monitoring, repeat imaging, and timely neurosurgical intervention in severe herpes simplex encephalitis complicated by malignant cerebral edema.

## Introduction

Herpes simplex encephalitis (HSE) is an inflammatory process incited by herpes simplex virus (HSV) infection in the nervous system through the viral induction of oxidative stress [1]. HSV-1 and HSV-2 are very common, and can infect patients and remain dormant for life. HSV-1 is predominantly transmitted orally with an incidence rate of over 55%. It is responsible for most cases of HSE in adults [1,2]. Most cases of HSV-1 infection are latent in the nervous system and show no symptoms. However, latent infections can be periodically reactivated to become symptomatic. Peripherally, this can manifest as periodic breakouts of oral sores due to reactivation of the virus along the trigeminal nerve, while central nervous system involvement may manifest as mild to severe neurologic complications including strokes and cognitive deficits [1,3,4]. Risk factors for reactivation include stress and being in an immunocompromised state. In symptomatic cases, HSV-1 infection can also cause encephalitis by infecting brain cells and causing vascular and neurologic changes. The virus induces necrotizing perivascular inflammation and edema most commonly in the temporal lobes and limbic areas including insular, cingulate, and inferolateral frontal cortices of the brain [5]. In most patients, HSV-1 encephalitis is mild, but can be fatal in a select few, especially if they are immunocompromised [6].

HSE diagnosis requires both clinical and objective findings. HSE presents with fever and a range of non-specific neurologic symptoms including stroke and seizure. HSE is best detected on magnetic resonance imaging (MRI) as diffuse hyperintensities in the brain due to tissue edema from inflammation [5]. HSE complications, such as cerebral edema, vascular changes, or hemorrhage are best detected with computed tomography (CT) which can reveal multifocal hyperdense lesions due to its acute nature. Lumber puncture (LP) with polymerase chain reaction (PCR) is an invasive procedure that can detect the presence of the virus in the cerebrospinal fluid (CSF). However, there are cases of encephalitis that are still not detected with these diagnostic modalities. In these cases, utilization of alternative modalities such as metagenomic next-generation sequencing (NGS) can be useful [7]. Although HSE can be fatal, early detection and management, and initiation of antiviral therapy can greatly improve the prognosis of this condition. Acyclovir is an effective therapy in patients with HSE [8]. Corticosteroids, even though not routinely used, can be useful in reducing inflammation in the brain [2,5]. Furthermore, some cases of HSE induce severe edema or hemorrhage in the brain, requiring timely surgical intervention to reduce intracranial pressures and alleviate the impact of mass effect from edema [9]. We report a case of HSE that rapidly worsened and developed malignant cerebral edema necessitating external ventricular drain (EVD) placement and an emergent decompressive hemicraniectomy.

## Case report

A 57-year-old woman with a history of hypertension, chronic alcohol and benzodiazepine use, and fibromyalgia was brought to the emergency department by family for a 3-day history of altered mental status, inability to perform activities of daily living, headache, vomiting, and myalgias. On presentation, she was afebrile and hemodynamically stable. Neurologic examination was notable for inattentiveness, confabulations, left visual field deficits, left facial weakness, and decreased sensation in the bilateral upper and lower extremities. Initial laboratory studies showed mild electrolyte abnormalities, a serum alcohol level of zero, and a urine drug screen positive for benzodiazepines. CT of the head without contrast showed no acute hypoattenuation and CT angiography of the head and neck demonstrated no vascular abnormality. MRI of the brain however demonstrated mild fluid-attenuated inversion recovery (FLAIR) hyperintense signal, along with mild restricted diffusion, appearing hyperintense on diffusion weighted imaging (DWI) with corresponding hypointesnity on the apparent diffusion coefficient (ADC) map involving the mesial right temporal lobe with extension into the insular cortex (Fig. 1, Fig. 2). Susceptibility-weighted imaging (SWI) demonstrated no evidence of hemorrhage. Empiric acyclovir (10 mg/kg every 8 hours) and high-dose thiamine were initiated.

**Fig. 1:**
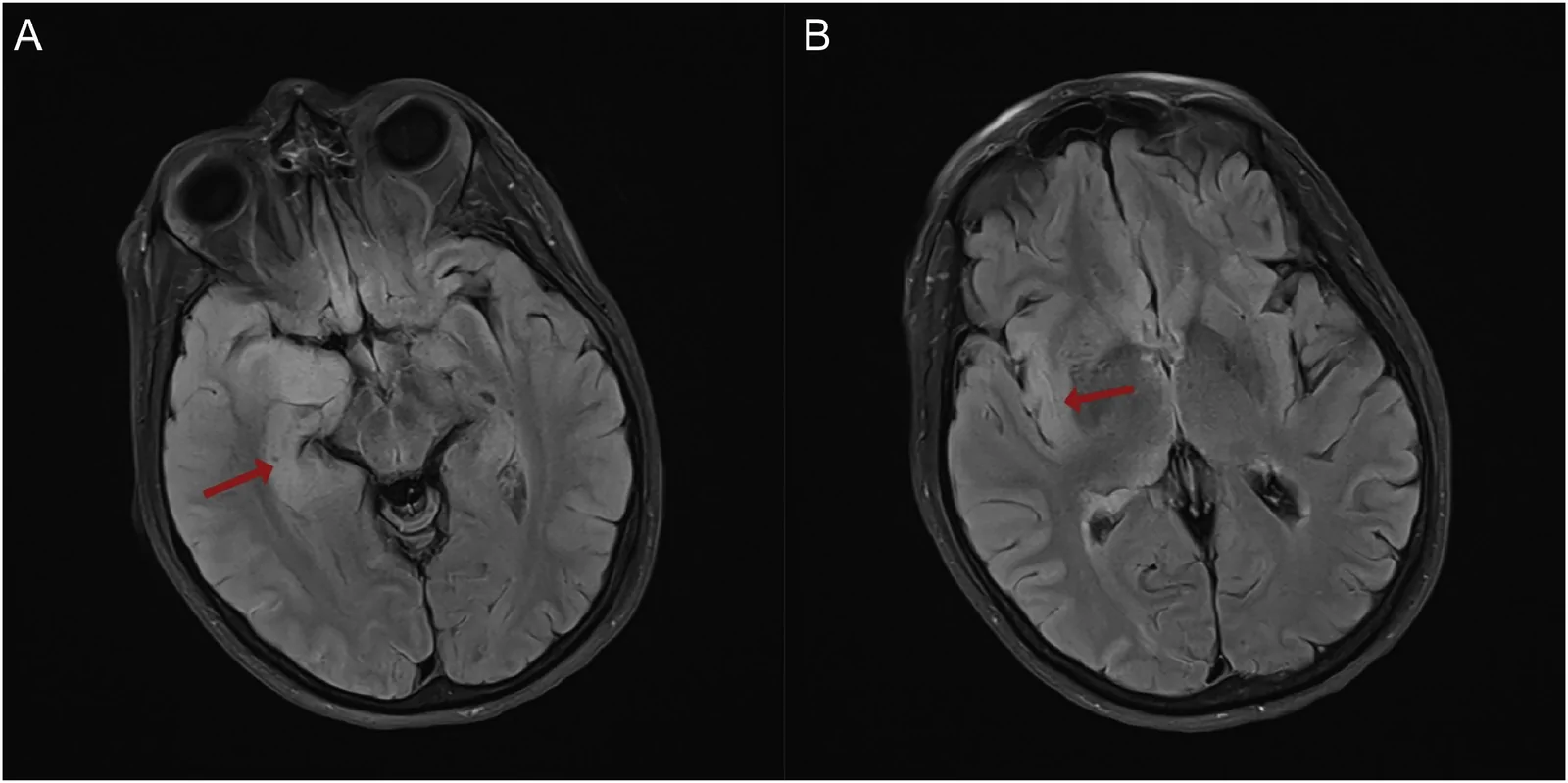
MRI brain (T2 FLAIR) without contrast showing mild hyperintense signal in the mesial right temporal lobe (A, red arrow), extending into the right insular cortex (B, red arrow).

**Fig. 2:**
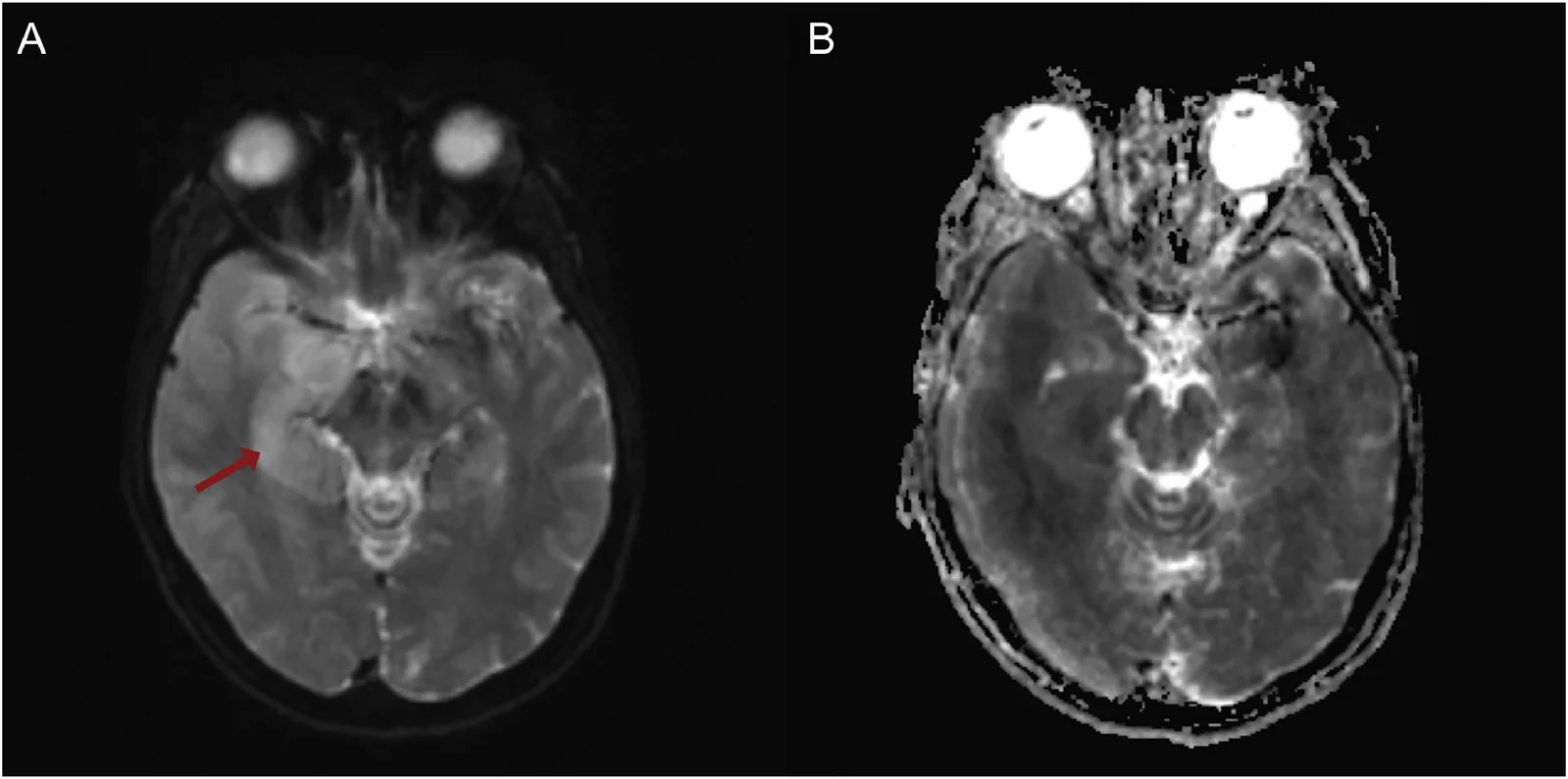
MRI brain (DWI [A] and ADC [B]) without contrast showing mild cytotoxic diffusion restriction involving the mesial right temporal lobe (red arrow).

On hospital day 2, she became febrile to 103.1°F, more somnolent, and developed subtle left-sided weakness. Repeat brain MRI 41 hours later demonstrated extensive hyperintense signal involving the right frontal, temporal, and parietal lobes, including the right insular cortex, right mesial temporal lobe, and right posteromedial thalamus, with associated mass effect (Fig. 3, Fig. 4). Although additional pathologies such as infiltrative neoplasm, postictal changes, or other encephalitic processes were considered, subsequent lumbar puncture revealed cerebrospinal fluid positive for HSV-1 with 271 nucleated cells, glucose 32 mg/dL, and protein 275 mg/dL. Additional studies for alternative viral, bacterial, fungal, and autoimmune etiologies were negative. Electroencephalography showed no seizures but demonstrated increased epileptogenicity in the right posterior temporal region, consistent with right hemispheric cortical dysfunction. Levetiracetam was started for seizure prophylaxis.

**Fig. 3:**
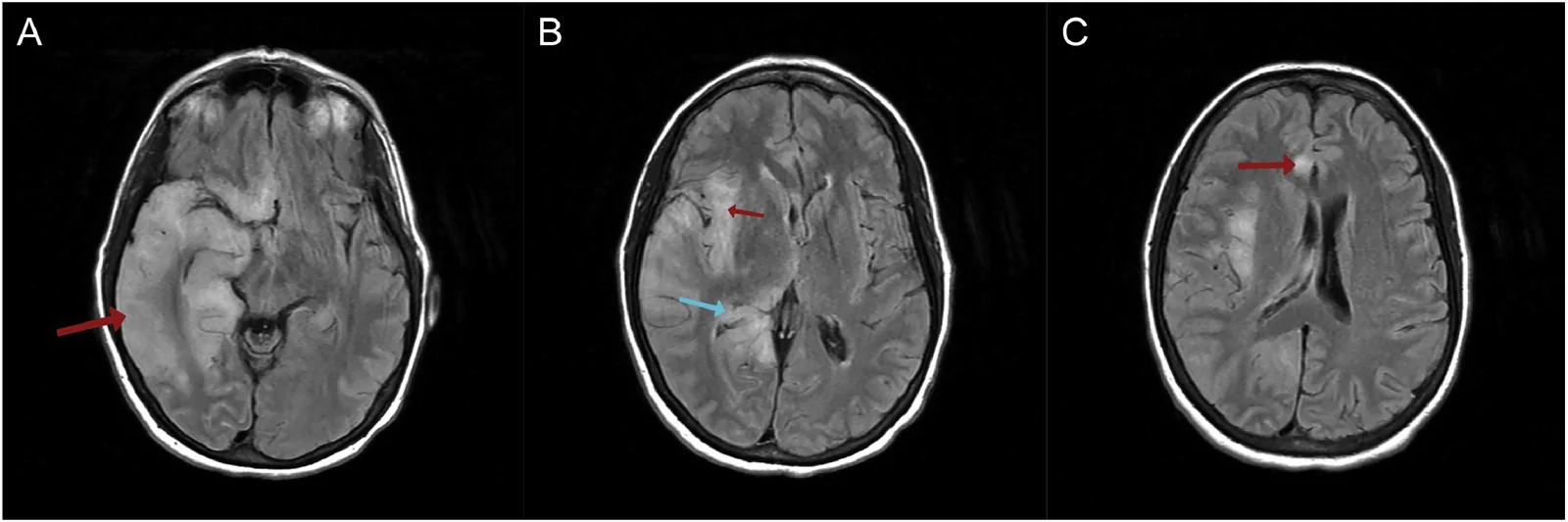
MRI brain (T2 FLAIR) without contrast showing hyperintense signal in the right temporal lobe (A, red arrow), right insular cortex (B, red arrow), right posteromedial thalamus (B, blue arrow), right frontal lobe (C, red arrow), and right parietal lobe (C).

**Fig. 4:**
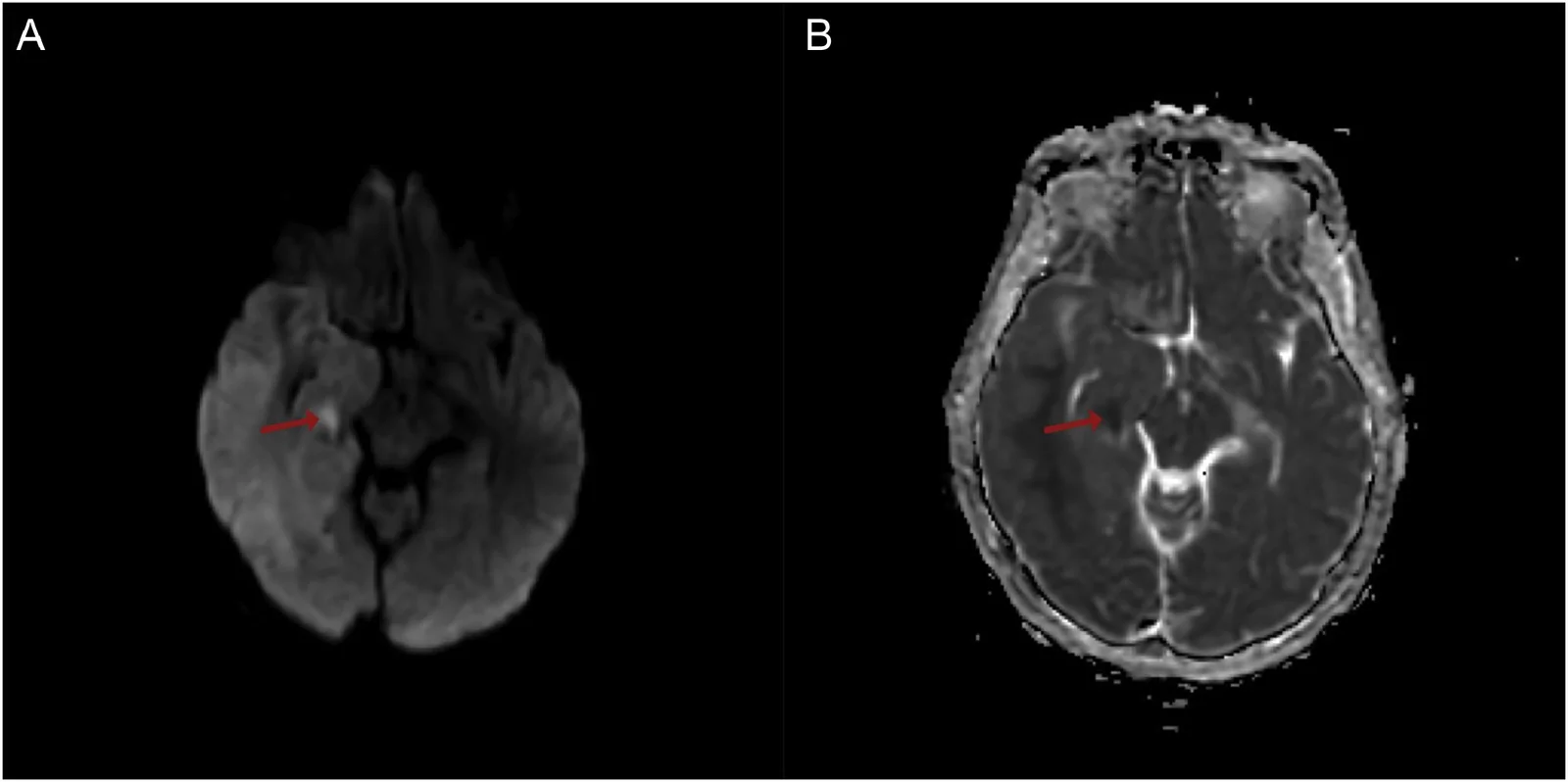
MRI brain (DWI [A] and ADC [B]) without contrast showing area of cytotoxic diffusion restriction involving the mesial right temporal lobe (red arrows).

On hospital day 3, she developed acute facial droop and left arm weakness. Repeat CT head without contrast demonstrated malignant edema involving the right temporal and frontal lobes with 11 mm of midline shift at the septum (Fig. 5). She was taken emergently to the operating room, where an external ventricular drain (EVD) was placed for intracranial pressure (ICP) monitoring.

**Fig. 5:**
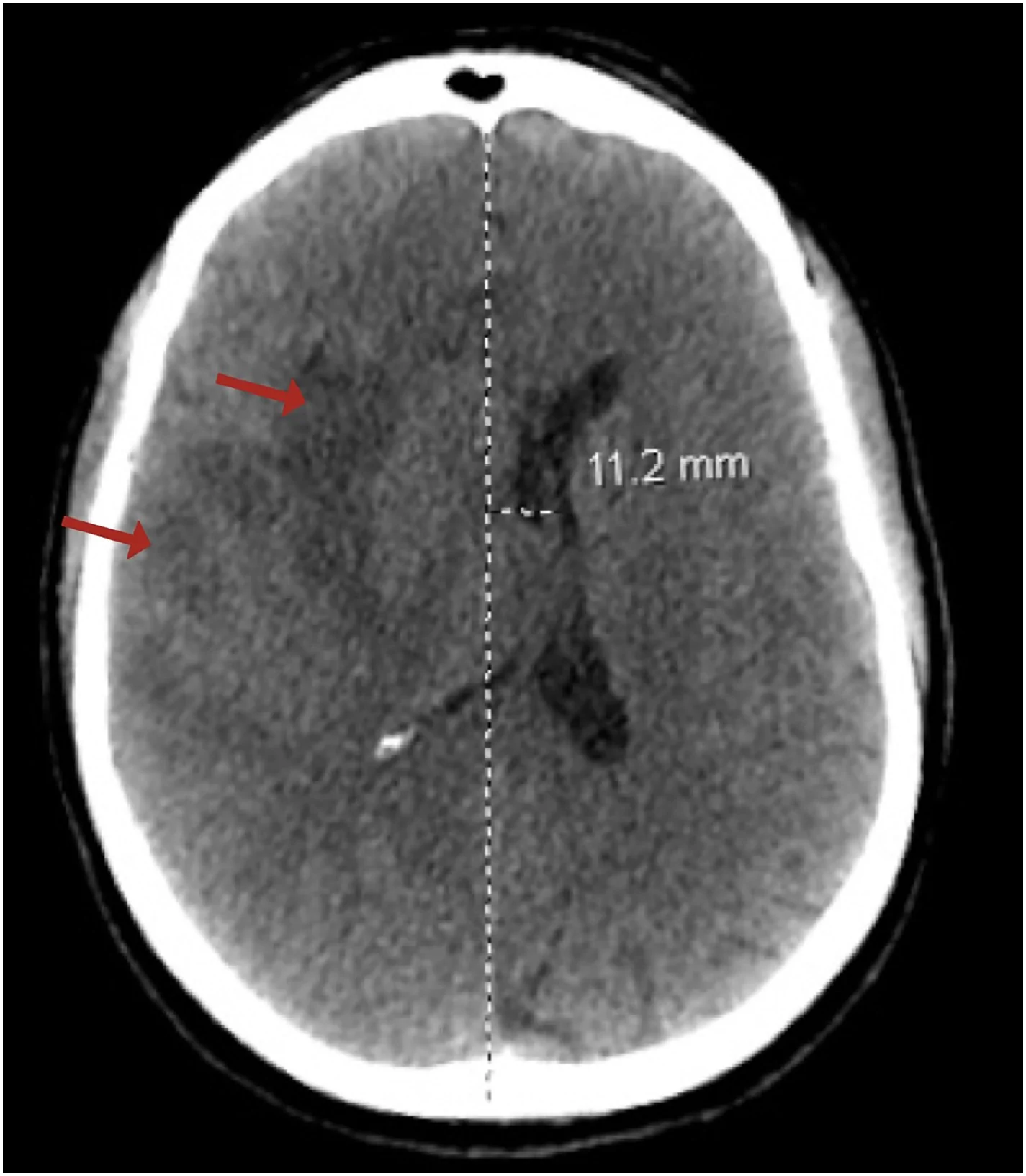
CT head without contrast showing extensive vasogenic edema within the right frontal and right temporal lobes (red arrows), with right cerebral sulcal effacement and worsening 11.2 mm midline shift at the septum pellucidum (dotted line). There is near-complete effacement of the right lateral ventricle with mass effect on the left and effacement of the third ventricle, without hydrocephalus, or uncal or subfalcine herniation.

Two days later, she experienced rapid exam changes with increased lethargy and left hemiparesis, prompting a CT head, which showed progressive malignant cerebral edema involving the right middle cerebral artery (MCA) territory with mass effect and worsening midline shift to 14 mm (Fig. 6). She subsequently underwent emergent right decompressive hemicraniectomy. Postoperative CT showed redemonstrated hypodensities in the MCA territory and improved mass effect, with midline shift decreased to 10 mm (Fig. 7).

**Fig. 6:**
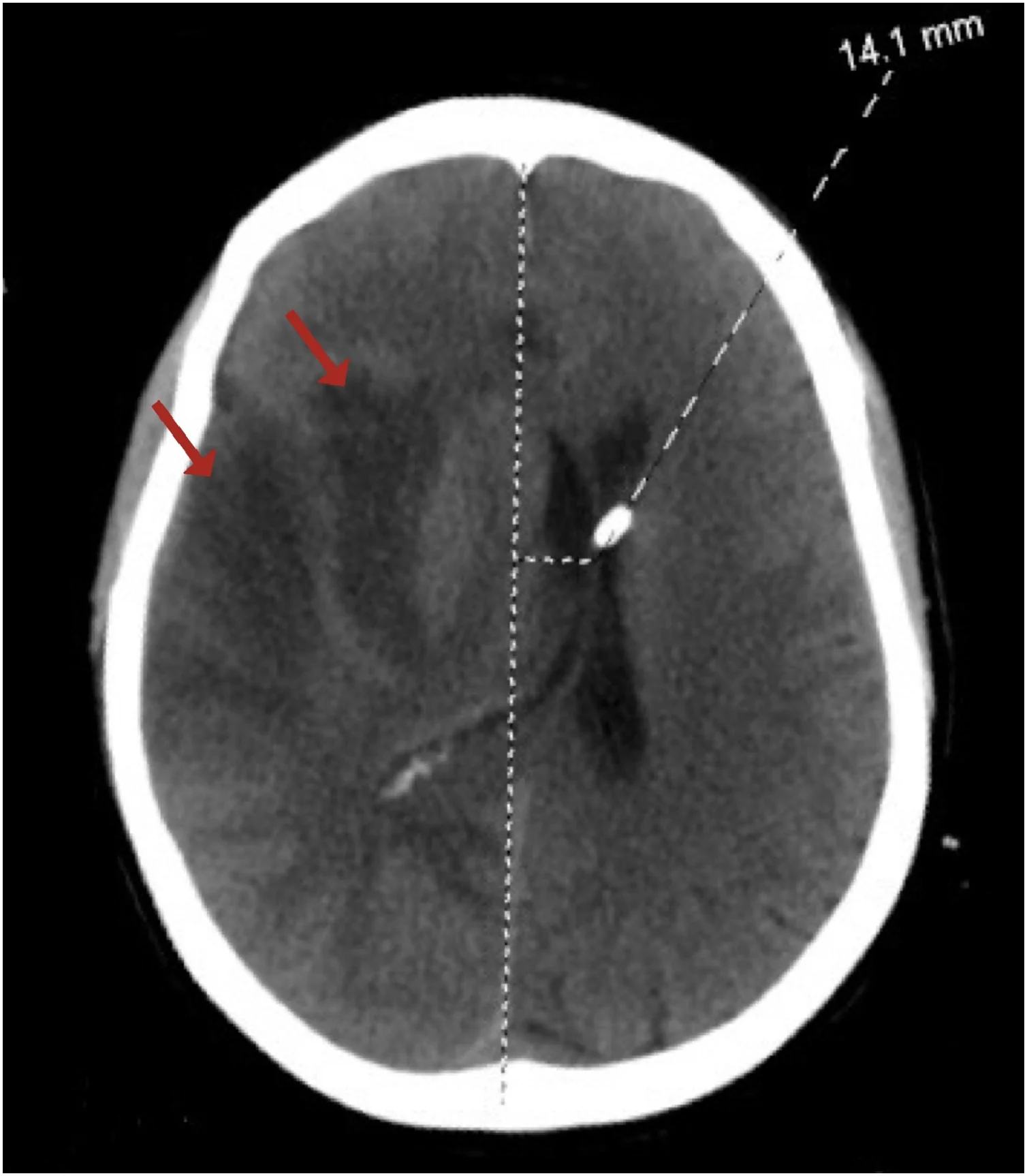
CT head without contrast post-EVD placement showing extensive vasogenic edema throughout the frontal, occipital, and temporal lobes (red arrows), with worsening 14.1 mm midline shift at the septum pellucidum (dotted line). The ventriculostomy shunt catheter is unchanged in position. There continues to be effacement of the right lateral ventricle, however, with re-expansion of the left lateral ventricle. There is no hydrocephalus, or uncal or subfalcine herniation.

**Fig. 7:**
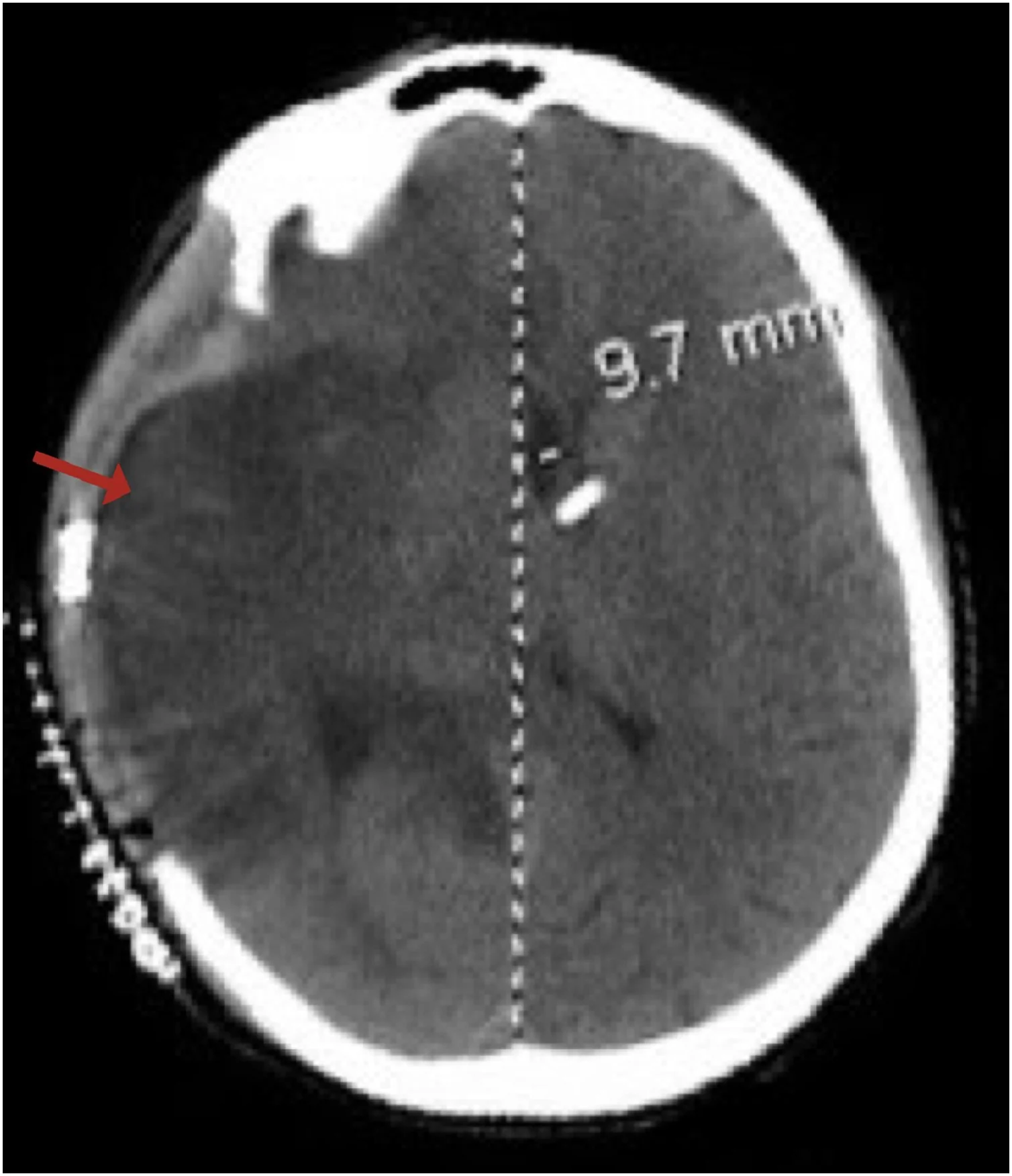
CT head without contrast post-right frontoparietal craniectomy showing redemonstrated hypodenities in the frontal, temporal, occipital lobes and the right MCA territory (red arrow). There is slightly improved midline shift, now measuring 9.7 mm from the septum pellucidum (dotted line). There is no hydrocephalus or transtentorial herniation.

Over the following days, the EVD was weaned and she improved to being alert and oriented to person, place, and time, reported no pain, and did not display any neurologic deficits. During this period, she remained intubated for hypoxic respiratory failure, but was successfully extubated with neurological recovery. She was then discharged to inpatient rehabilitation. Three weeks later, repeat CT head demonstrated marked improvement in cerebral edema with improving right temporal hypodensities and minimal mass effect (Fig. 8). Her neurologic examination remained non-focal, and she underwent cranioplasty two months later.

**Fig. 8:**
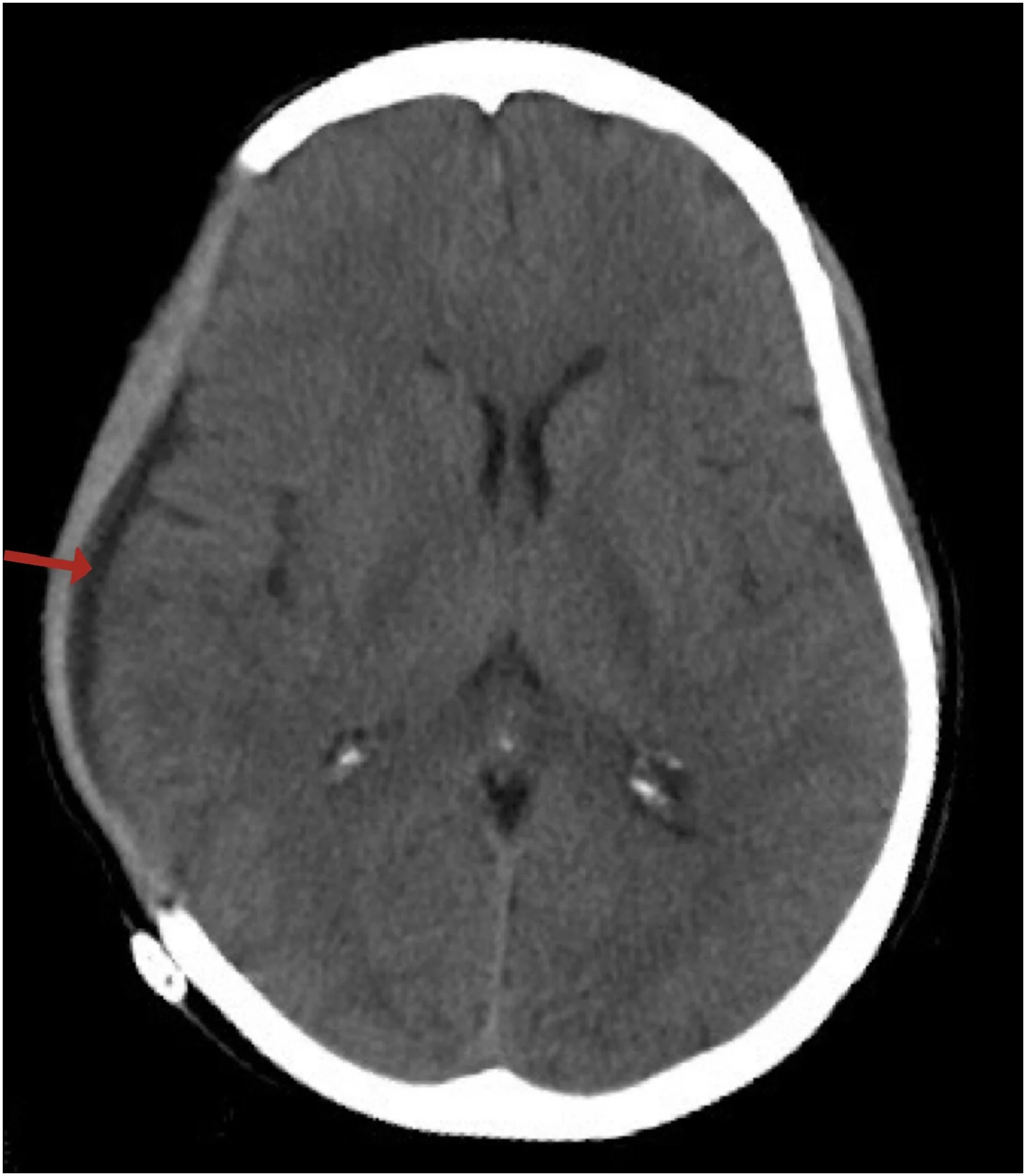
CT head without contrast showing significantly improved cerebral edema with improving right temporal hypodensities and minimal mass effect. There is a trace simple appearing effusion underlying the cranioplasty flap (red arrow).

## Discussion

HSE is a severe form of brain inflammation that can lead to a range of neurological complications, including altered mental status and neurologic deficits. While small petechial hemorrhages are relatively common in HSE, the development of large intracerebral hematomas or edema, especially requiring surgical intervention, is rare. Only a small number of such cases have been reported; in these cases, treatment with acyclovir was started promptly [10]. Although the exact cause is not fully understood, it is believed that inflammation from encephalitis may weaken blood vessels (vasculitis) or increase ICP, leading to vessel rupture [1].

Because HSE is a rapidly progressing encephalitis, early treatment is essential. Acyclovir should be initiated immediately to limit viral replication and reduce brain damage [5,10]. Delays in treatment significantly increase the risk of long-term neurological deficits or death. The Infectious Disease Society of America recommends 2–3 weeks of intravenous antiviral therapy depending on the patient’s clinical response. However, diagnosing encephalitis can be difficult, as some patients may present with atypical symptoms or normal early CSF findings [7]. For this reason, clinicians are encouraged to repeat testing when encephalitis is suspected and to begin treatment even before confirmation.

As encephalitis progresses, patients must be closely monitored for worsening neurological status. The appearance of new symptoms, such as altered mental status, focal neurologic deficits, or pupil changes, should raise concern for complications like increased ICP or cerebral edema [10]. In such cases, neuroimaging becomes critical to evaluate disease progression and guide treatment decisions.

Management of severe encephalitis includes controlling brain swelling and ICP. Medical approaches may involve hyperventilation, corticosteroids, and hyperosmolar therapies such as mannitol or hypertonic saline. Although the use of corticosteroids in encephalitis remains debated due to concerns regarding increased viral replication, evidence suggests they may help reduce inflammation and edema when used after antiviral therapy has begun [5,9]. Similarly, hyperosmolar therapy, while not specific to encephalitis, is often applied based on principles from traumatic brain injury management to help reduce brain swelling.

In more severe cases of encephalitis, life-threatening changes like cerebral edema, increased ICP, or hemorrhage occur in the brain [10], necessitating surgical intervention to reduce mortality. Procedures such as decompressive craniectomy or temporal lobectomy can relieve pressure on the brain and prevent further damage [9]. Although data is limited, existing evidence supports consideration of surgical decompression when medical management fails. Patients who undergo timely surgical intervention may recover with minimal or no lasting neurological deficits [9]. In our patient, decompressive hemicraniectomy greatly improved her clinical presentation, resulting in no neurologic deficits three months later.

## Conclusion

In summary, encephalitis, particularly HSE, can lead to serious complications, including rare but dangerous intracerebral edema. Early recognition, prompt antiviral treatment, and close monitoring are essential to improving outcomes. When complications arise, aggressive medical management and, when necessary, surgical intervention should be considered to prevent further neurological deterioration.

## Author contributions (ICMJE criteria)

**Evans Konney:** Conception and design of the study; acquisition and analysis of data; drafting of the manuscript; critical revision of the manuscript for important intellectual content; final approval of the version to be published. **Jonathan R. Forrest:** Conception and design of the study; acquisition and analysis of data; drafting of the manuscript; critical revision of the manuscript for important intellectual content; final approval of the version to be published. **Tapan Thaker:** Conception and design of the study; acquisition and analysis of data; drafting of the manuscript; critical revision of the manuscript for important intellectual content; final approval of the version to be published. **Gnan Thakore:** Conception and design of the study; acquisition and analysis of data; critical revision of the manuscript for important intellectual content; final approval of the version to be published.

All authors meet ICMJE criteria for authorship and have approved the final manuscript.

## Patient consent

Informed consent was obtained from the patient for publication of this case report and accompanying images.
